# Comparison of Serum Trefoil Factor-3 to Endoscopy in Diagnosing *Helicobacter Pylori* Associated Gastric Ulcer

**DOI:** 10.31557/APJCP.2020.21.7.2149

**Published:** 2020-07

**Authors:** Ragaa A Ramadan, Moyassar A Zaki, Said A Ooda, Hosam M Abo Khalifa, Wafaa S Ragab

**Affiliations:** 1 *Chemical Pathology, Medical Research Institute, Alexandria University, Egypt. *; 2 *Departments of Internal Medicine, Medical Research Institute, Alexandria University, Egypt. *; 3 *Edfina Hospital, Behira, Egypt. *

**Keywords:** H.pylori infection, gastric ulcers, Trefoil factor-3

## Abstract

**Background and aim of the work:**

*Helicobacter pylori*-associated gastric ulcer (*H.pylori*-GU) is a serious condition, not only because *H.pylori* is identified as a grade 1 carcinogen but also because GU is a precancerous condition. Identification and treatment of *H.pylori*-GU may prevent the sequential progression of dysplasia to carcinoma. Trefoil factor 3 (Tf3) has been implicated in gastric mucosal repair. We compared serum Tf3 to gastric endoscopy in diagnosing *H.pylori*-GU.

**Subjects and methods:**

The study included eighty patients suffering from *H.pylori* induced gastritis, forty of which presented with GU. Gastric endoscopy with slide urease test was used to diagnose *H.pylori*-GU. Serum Tf3 level was determined using an enzyme immunoassay in all patients as well as thirty healthy volunteers.

**Results:**

Serum Tf3 showed a significant stepwise decrease among the studied groups. It was significantly lower in patients compared to the control group (p<0.001). Furthermore, it was lower in those with GU compared to those without GU (p=0.023). Based on a receiver operating characteristic curve generated cut off value of 2.4 ng/mL, the diagnostic performance of serum Tf3 as a biomarker of *H.pylori*-GU revealed a diagnostic specificity of 42.5%, sensitivity of 67.5%, positive and negative predictive values of 54% and 56.67% respectively.

**Conclusion:**

Although serum Tf3 showed significant variation in *H.pylori*-GU, further studies are warranted to confirm its role in the pathogenesis of gastric ulcers.

## Introduction

Gastric ulcer (GU) is a precancerous pathology with an increased risk of carcinogenesis (Lv et al., 2011). The management of peptic ulcer diseases, namely screening and therapy, has been extensively studied. Clinical research focused on the cost-effective value of diagnostic and pharmacological interventions of such high-risk patients. Some gastrointestinal societies presented international recommendations and evidence-based guidelines regarding precancerous gastric lesions. *Helicobacter pylori* (*H.pylori*) infestation is a core issue in this context and its serological diagnosis and complete eradication are some of the high levels of evidence grades to prevent dysplasia and carcinoma. (Dinis-Ribeiro et al., 2012).

*H.pylori* is one of the common infestations with a prevalence of 50.8% in the developing countries and 34.7% in the developed countries. (Zamani et al., 2018) Although *H.pylori* infection can be asymptomatic, yet it can be associated with chronic gastritis, gastric or duodenal ulcers, gastric adenocarcinoma, and type-B low-grade mucosal-associated lymphoma. It is also involved in hematologic and autoimmune disorders, insulin resistance, and metabolic syndrome (Sabbagh et al., 2019) 

Certain points have to be addressed regarding endoscopy in *H.pylori* gastritis. First although conventional endoscopy cannot reliably diagnose H. pylori gastritis, atrophy, or intestinal metaplasia, magnifying endoscopy has resolved these limitations (Redéen et al., 2003). Second, the biopsy protocol regarding the number and site of gastric sampling is controversial because of the multifocal nature of *H.pylori*. Lastly, the agreement between pathologists is variable. (Dinis-Ribeiro et al., 2012). Several biomarkers have been studied in *H.pylori* infection, among which is the trefoil factor family of peptides.

Trefoil factors (Tfs) comprise a unique family of abundant gastrointestinal (GI) peptides with a distinct three-loop structure formed by a highly conserved motif of cysteine disulfide bonds. They are extremely stable against proteolytic digestion and acid degradation. The trefoil factor family consists of three members: Tf1 (breast cancer-associated peptide pS2), Tf2 (spasmolytic polypeptide), and Tf3 (intestinal trefoil factor). Tf3 is predominantly expressed in the goblet cells of the small intestine and colon (Taupin and Podolsky, 2003). 

Trefoil factor 3 has a key role in mucosal defense and restitution in the gastrointestinal mucosa. (Taupin and Podolsky, 2003) It has an anti-apoptotic and anti-anoikic effect on intestinal epithelial cells. Moreover, it contributes to the stabilization and maintenance of the intestinal epithelial barrier function by simulating the recovery of tight junction proteins. (Aamann et al., 2014) An emerging role for Tf3 has been documented in oncogenic transformation, and metastatic extension of solid tumors, such as prostate (Abou-Ouf et al., 2019) and breast cancers. (Al-Salam et al., 2019)

The suitability of blood-based biomarkers in diagnosing gastric metaplasia was tackled in several studies. For instance, Lee et al (2017) study revealed a close correlation between serum pepsinogen and upper gastrointestinal endoscopic findings in seropositive and seronegative *H.pylori* subjects. Moreover, they deduced that serum Tf3 outperformed pepsinogen in screening for gastric cancer. (Lee JY et al., 2017) We questioned the use of serum Tf3 as a diagnostic tool in *H.pylori* associated gastric ulcer and compared its diagnostic performance to the gold standard tool (endoscopy).

## Materials and Methods

Eighty patients infected with *H.pylori* were recruited from the hepatology unit of the Internal Medicine Department during the period from July 2016 till July 2017. *H.pylori* infestation was diagnosed by a stool-based Antigen test (On-Site H. Pylori Ag Rapid Test, CTK Biotech, San Diego, CA). Clinical examination was done to all the patients with stress on symptoms of gastritis such as heartburn and dyspepsia. Exclusion criteria included those below 18 years, excessive intake of alcohol, recent intake of antibiotics, and proton pump inhibitors within the past month. Gastrointestinal malignancy, drug-induced peptic ulcers, pregnancy, and lactation were also excluded.

Based on upper gastrointestinal endoscopy (Olympus GIF- XQ 240), patients were classified into those with and without GU. All the gastric biopsies were taken from the antrum and/or the corpus of patients. Gastric biopsy was used to perform the rapid slide urease test (helicotecUT plus kit) as a confirmatory test for *H.pylori* infestation. All H. pylori-infected cases had their treatment stopped for one week before endoscopy to avoid false sampling from the migration of H. pylori proximally in the stomach from treatment. Thirty healthy volunteers were enrolled from the outpatient clinics of the Institute to serve as age and gender-matched control group. 

Informed consent was obtained from all study participants. Study design and procedures were following the ethical standards of the Ethical Committee of Medical Research Institute, Alexandria University (IORG#: IORG008812), and with the 1964 Helsinki declaration and its later amendments.

Five milliliters whole venous blood were drawn from each subject following an overnight fasting period. The blood sample was collected in both serum and EDTA vacutainer tubes where the latter was used for the determination of hemoglobin, red cell, total leucocytic, and platelet counts using an automated 3 part cell counter (Mindray Inc., Shenzhen, China). The serum obtained following centrifugation of the serum vacutainer tube was used for the determination of serum creatinine, albumin and iron levels as well as total iron-binding capacity (TIBC), in addition to the activities of aspartate and alanine aminotransferases.

Analyses were conducted on the Olympus AU400 clinical chemistry analyzer (Beckman Coulter Inc., Brea CA, USA). Ferritin was determined using a two-site immuno chemiluminometric assay on the Immulite-1000 immunoassay analyzer (Siemens Healthineers Inc., Tarrytown NY, USA). The rest of the sample was stored at -20°C till the time of assay of serum Tf3 level that was determined using an enzyme immunoassay (RD191160200R-Biovendor Laboratorni Medicina a.s., Modric, Czech Republic, EU) according to the manufacturer’s instructions. 

*Statistical analysis*

Statistical analysis was done using the IBM SPSS version 20 (SPSS, Inc., Chicago, IL, USA). Qualitative data were described using numbers and percentages. The distributions of quantitative variables were tested for normality using the Kolmogorov-Smirnov test, Shapiro-Wilk test, and D’Agstino test. Descriptive data were presented as mean and standard deviation for normally distributed parameters, while median and range were resorted to non- normally distributed data. The comparison of variables between groups was done for normally distributed data using analysis of variance (ANOVA) and independent samples t-test, while in non-normally distributed data Kruskal-Wallis and Mann-Whitney tests were used. The patient groups were compared using the Chi-square test. When more than 20% of the cells have expected count less than 5, correction for chi-square was conducted using either Fisher’s exact test or Monte Carlo test. A p-value of less than 0.05 was considered statistically significant. For choosing the best cut off value, the receiver operator characteristic (ROC) curve was generated and Youden’s index was calculated (28). The diagnostic performance of serum Tf3 to diagnose *H.pylori* GU was compared to the golden standard tool (endoscopy).

## Results

The study included 30 healthy volunteers and 80 patients infected with *H.pylori*. The patients were divided into 40 cases without GU (group-A), and 40 cases with GU (group-B). The studied groups were age and gender-matched (p˃0.05) (Table 1).

There was no difference in the median values of Tf3 between males and females among the studied groups. 

When compared to the control group, *H.pylori* infected patients had a significant decrease in the studied hematological parameters including hemoglobin, red cell, total leucocytic and platelet counts. Anemia was prevalent in 88.8 % of cases in the whole patients’ group; 52.5 % of which was hypochromic microcytic anemia while the rest was normochromic normocytic. There was no predominant type of anemia in patients’ groups; hypochromic microcytic anemia was found in 55% and 50% of patients in group-A and group-B respectively. Although there was a significant reduction of the platelet count in patients’ group compared to the control group, only 10 cases (12.5%) had thrombocytopenia. 

Serum albumin and iron levels as well as TIBC were significantly lower in patients compared to the control group, whereas AST activity was higher in patients compared to the control group. Meanwhile, there was no significant difference between groups A and B regarding serum ferritin levels. 

Serum Tf3 showed a significant stepwise decrease among the studied groups. It was significantly lower in patients compared to the control group (p<0.001). Furthermore, it was lower in those with GU (group B) compared to those without GU (group A) (p=0.023) (Table 2). The diagnostic performance of serum Tf3 was compared to the golden standard tool (upper GIT endoscopy) for diagnosing GU. A ROC curve generated cut off value for serum Tf3 of 2.4 ng/mL gave a diagnostic sensitivity of 67.5% and specificity of 42.5 % with positive and negative predictive values of 54 % and 65.67 % respectively, an overall test accuracy of 55% (Table 3).

**Table 1:** Demographic Data of the Studied Groups

Items	Control group (n=30)	Group (A) (n=40)	Group (B) (n=40)	Test of sig.	*P*-value
Age; median (years)	45 (32–70)	46 (20–69)	49 (22–74)	H = 0.002	0.999
Gender; n (%)					
Males	12 (40%)	20 (50%)	18 (45%)	χ2 = 0.697	0.706
Females	18 (60%)	20 (50%)	22 (55%)		

Group A, patients without gastric ulcers; Group B, patients with gastric ulcers; p, p value for comparing between the two studied groups; t, Student t-test; χ^2^, Chi square test

**Table 2:** Selected Hematological and Biochemical Parameters among the Studied Groups

Parameter	Control group	Patient group (n=80)		p1	p2
	(n=30)	Group (A)	Group (B)		
		(n=40)	(n=40)		
Trefoil factor-3 (ng/mL)	7.4 (0.33–13.15)	2.19 (1.42–6.96)	1.81 (1.08–7.64)	<0.001	0.023
Hemoglobin (gm/dl)	13.54 ± 1.28	11.44 ± 1.37	11.18 ± 1.56	<0.001	0.426
RBCs count (x10^6^) (/cmm)	4.94 ± 0.37	4.39 ± 0.38	4.34 ± 0.50	<0.001	0.636
TLC (x103) /cmm	6.99 (4.0–8.6)	4.75 (3.45 – 9.10)	5.3 (3.14 – 10.90)	<0.001	0.143
Platelet count (x10^3^) (/cmm)	306 (207–410)	206 (179–306)	190 (100–199)	<0.001	0.718
Creatinine (mg/dL )	0.89 ± 0.12	0.96 ± 0.26	0.86 ± 0.15	0.092	0.053
Iron (μg/dL)	76 ± 19	56 ± 21	57 ± 22	<0.001	0.798
TIBC (μg/dL)	287 ± 23	254 ± 31	262 ± 58	0.004	0.414
Ferritin (μg/L)	113 ± 28	134 ± 90	144 ± 63	0.156	0.358
Albumin (g/dL)	4.2 ± 0.2	3.5 ± 0.6	3.6 ± 0.4	<0.001	0.397
AST (U/L)	22 (15–33)	36 (10–63)	30 (11–125)	0.001	0.942
ALT (U/L)	20 (10–37)	16 (2–41)	17 (4–38)	0.05	0.278

Group A, patients without gastric ulcers; Group B, patients with gastric ulcers; ALT, Alanine aminotransferase, AST, Aspartate aminotransferase, RBCs, Red blood cells; TIBC, Total Iron Binding Capacity; TLC, Total leucocytic count; p1, p-value for comparing between the three studied groups; p2, p-value for comparing between group (A) and group (B); n, number of studied subjects in each group; F, ANOVA test; H: Kruskal Wallis test; SD, standard deviation.

**Table 3:** Diagnostic Performance of Serum Tf3 in Cases Presenting with Gastric Ulcers versus Cases without Gastric Ulcers

Item	Cut off value (ng/mL)	Group A	Group B	Sensitivity	Specificity	PPV	NPV	Accuracy
		(n=40)	(n=40)					
Tf3	>2.4	17	13	67.5	42.5	54	56.67	55
(ng/ml)	≤2.4	23	27					

Group A, patients without gastric ulcers; Group B, patients with gastric ulcers; NPV, Negative predictive value; PPV: Positive predictive value

## Discussion

Gastrointestinal endoscopy and the acquisition of tissue samples are mandatory for the management of various diseases of the digestive system. Peptic ulcer diseases and *H.pylori* infestation are among the main indications for biopsies in upper endoscopy. Guidelines are developed to rationalize the use of endoscopy used to avoid its overutilization. This is triggered by the high financial burden of healthcare costs especially in developing countries as well as the complications and the invasive nature of the endoscopy. (Dinis-Ribeiro et al., 2012)

Non-invasive lab biomarkers are attractive tools that can serve as a preliminary step to identify and triage those requiring endoscopy. We questioned the use of serum Tf3 as a diagnostic tool in *H.pylori* associated GU and compared its diagnostic performance to the golden standard tool. 

Trefoil factor-3 is a key player in gastric mucosal integrity and homeostasis. In *H.pylori* infestation there is a continuous state of gastric insult ranging from chronic active gastritis till gastric ulcer. In the current work, we observed a stepwise decrease in serum Tf3 levels from healthy volunteers with normal intact gastric mucosa (7.4± 2.15 ng/ml) to *H.pylori* subjects without GU (2.60±1.39 ng/ml) then finally in *H.pylori* associated gastric ulcer subjects (2.07± 1.09 ng/ml). On the other hand, Kaise et al (2013) reported serum levels of Tf3 in H. pylori seropositive and seronegative subjects to be (5.85 ± 3.93 ng/ml) and (5.27 ± 2.38 ng/ml) respectively. Although serum Tf3 levels were quite similar in the absolute value, yet the difference showed to be significant (p=0.002). Srivastava et al (2015) reported a value of 0.85 (IQR 0.7–1.2) ng/ml in healthy volunteers. 

Infection with *H.pylori* elicits the release of a wide array of proinflammatory and immunoregulatory cytokines. Their pattern is heterogeneous based on several factors such as disease status and bacterial genotype. For instance, Vinagre et al showed that the gastric concentrations of IFN-γ and IL-12 were higher, while IL-4 and IL-10 were lower in H. pylori subjects compared to healthy individuals. They also showed differential expression of cytokines according to the various bacterial strains. (Vinagre et al., 2018) Similarly, Milic et al reported an altered release of serum cytokines in *H.pylori* patients namely an increase in IL-6 and TNF concentrations and a decrease in TGF-β and IL-17A. (Milic et al.,2019) The regulatory mechanisms of Tf3 and even the existence of its related receptor are still unresolved issues. Yet several routes have been postulated to explain its mediated actions. Tf3 promotes the activation of a number of cytokine-dependent pathways like mitogen activated protein kinase (MAPK), beta catenin, and/or epidermal growth factor receptor (EGFR). (Belle et al., 2019) We can assume that the interaction of the distorted cytokines milieu of *H.pylori* infection with the Tf3 related pathways could lead to the observed reduction in Tf3 levels compared to the healthy group. While the significant decrease of Tf3 in GU cases could point out to an impairment of repair and restitution of gastric epithelium. 

Serum Tf3 was questioned as a prognostic biomarker in other GIT pathologies. Srivastava et al (2015) reported a stepwise increase of serum Tf3 from healthy volunteers to ulcerative colitis patients with mucosal healing to those without mucosal healing. They deduced a serum Tf3 level of <1.27 ng/ml for detecting patients with mucosal healing with a diagnostic sensitivity of 70% and a specificity of 68%. On the other hand, Eder et al (2017) failed to prove Tf3 as an effective biomarker of mucosal healing in patients with Crohn’s disease treated with anti-TNF-alpha antibodies. Their study showed no significant correlation between Tf3 and the simple endoscopic score for Crohn’s disease, nor with fecal calprotectin. Interestingly, they noted a decrease in the level of serum Tf3 in patients with failed mucosal healing. This finding may be comparable to our observation of decreased Tf3 in GU group denoting their impaired healing.

Results must be interpreted cautiously. First, there are distinctions in the pathophysiology of various GIT diseases. Second, the gastric and the intestinal epithelium are histologically variable. Third, the differential expression of Tf3 which is more predominant in the intestinal goblet cells compared to the gastric epithelium. Finally, our patients did not receive any medication, while subjects in Srivastava’s and Eder studies were on anti-inflammatory or immunosuppressive medications.

Similar to Srivastava et al 2015, we noted the absence of statistical difference in Tf3 according to gender. Biological variations in Tf3 present a major limitation. Samson (2013) calculated a serum reference interval of Tfs (0.091–0.25 nmol/L) and pointed to the large inter-individual variation of Tfs in his work. Tf3 exists in different molecular forms and it was eluted with sizes ranging from monomers, dimers, and even larger molecules. Moreover, commercial assays lack standardization which hampers comparison of research results and evaluation of clinical efficacy of Tf3. But still, research is conducted on in measuring Tf3 in different body matrices such as stool and urine. (Heitkemper et al., 2018).

Despite the aforementioned limitations in measurements of Tf3, it is still a promising diagnostic and therapeutic tool in a wide range of diseases involving the mucous membrane. Clinical trial for the use of Tf3 enema for treatment of inflammatory bowel disease showed acceptable tolerance but unfortunately, it had no added value on the routine therapeutic strategy. (Mahmood et al., 2005) The immunohistochemical staining test for Tf3 is applied as a biomarker of intestinal metaplasia in the Cytosponge test which is gaining attention in screening for Barrette oesophagus and oesophageal adenocarcinoma. (Offman et al., 2018).

Infection with *H.pylori* is linked to several extra-gastroduodenal effects such as iron deficiency anemia and immune thrombocytopenic purpura. 88.8% of our *H.pylori* patients had anemia and 52.5 % of them were of the hypochromic microcytic type. Anemia can be attributed to iron loss in hemorrhagic gastritis, impaired iron absorption due to reduced gastric acidity as well as interference with iron acquisition from holotransferrin. (Tsay and Hsu, 2018) Immune thrombocytopenia is described in *H.pylori* infestation, but its mechanism is poorly understood. In our study, 12.5% had thrombocytopenia, yet this can not be solely attributed to *H.pylori* infection. One of the most accepted explanations is the molecular mimicry between H. pylori antigens and platelets. (Marques et al., 2019) 

Several issues remain unrevealed including the identification of the trefoil peptide receptor(s) which remains unknown even though trefoil peptides are biologically active and can trigger intracellular signaling mechanisms. Further experimental studies are needed to determine the therapeutic usefulness of Tf3 in improving the healing process following GU development. 

In conclusion, the present study demonstrates, to the best of our knowledge, one of the few studies exploring the association of serum Tf3 with H. pylori-related gastric ulcer development. Although endoscopy outperformed serum Tf3 in that context yet still further studies are needed for Tf3 on a tissue-based level to help in understanding its exact role in the pathophysiology of *H.pylori* associated gastric ulcers and increased rates of incomplete *H.pylori* eradication. 
